# Strong nonlinear terahertz response induced by Dirac surface states in Bi_2_Se_3_ topological insulator

**DOI:** 10.1038/ncomms11421

**Published:** 2016-04-26

**Authors:** Flavio Giorgianni, Enrica Chiadroni, Andrea Rovere, Mariangela Cestelli-Guidi, Andrea Perucchi, Marco Bellaveglia, Michele Castellano, Domenico Di Giovenale, Giampiero Di Pirro, Massimo Ferrario, Riccardo Pompili, Cristina Vaccarezza, Fabio Villa, Alessandro Cianchi, Andrea Mostacci, Massimo Petrarca, Matthew Brahlek, Nikesh Koirala, Seongshik Oh, Stefano Lupi

**Affiliations:** 1 INFN and Dipartimento di Fisica, Università di Roma ‘La Sapienza', Piazzale A. Moro 2, I-00185 Roma, Italy; 2 Laboratori Nazionali di Frascati—INFN, Via E. Fermi, 40, I-00044 Frascati, Italy; 3 INSTM Udr Trieste-ST and Elettra—Sincrotrone Trieste S.C.p.A, Area Science Park, I-34012 Trieste, Italy; 4 INFN and Dipartimento di Fisica, Università di Roma ‘Tor Vergata', viale della Ricerca Scientifica 1, I-00133 Roma, Italy; 5 INFN and Dipartimento S.B.A.I., Università di Roma ‘La Sapienza', Piazzale A. Moro 2, I-00185 Roma, Italy; 6 Department of Physics and Astronomy Rutgers, The State University of New Jersey, 136 Frelinghuysen Road, Piscataway, New Jersey 08854-8019, USA

## Abstract

Electrons with a linear energy/momentum dispersion are called massless Dirac electrons and represent the low-energy excitations in exotic materials such as graphene and topological insulators. Dirac electrons are characterized by notable properties such as a high mobility, a tunable density and, in topological insulators, a protection against backscattering through the spin–momentum locking mechanism. All those properties make graphene and topological insulators appealing for plasmonics applications. However, Dirac electrons are expected to present also a strong nonlinear optical behaviour. This should mirror in phenomena such as electromagnetic-induced transparency and harmonic generation. Here we demonstrate that in Bi_2_Se_3_ topological insulator, an electromagnetic-induced transparency is achieved under the application of a strong terahertz electric field. This effect, concomitantly determined by harmonic generation and charge-mobility reduction, is exclusively related to the presence of Dirac electron at the surface of Bi_2_Se_3_, and opens the road towards tunable terahertz nonlinear optical devices based on topological insulator materials.

## Abstract (web summary)

The terahertz response of topological insulator surface states, in which relativistic electrons are protected from backscattering, possesses potential optic and plasmonic applications. Here, the authors demonstrate a nonlinear absorption response of Bi_2_Se_3_ to terahertz electric fields.

## Graphical abstract

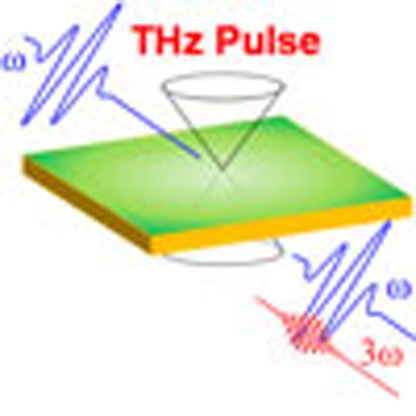

Nonlinear optical phenomena have a crucial importance in modern physics giving rise to fundamental applications such as coherent control of excitations in condensed matter, and harmonic generation and frequency conversion in optically active materials^1^,^2^,^3^. In this case, the use of materials whose electromagnetic response can be fully controlled by an applied radiation field plays a fundamental role in ultrafast electromagnetic pulse generation and shaping^4^. These nonlinear optical properties, earlier discovered and studied in the visible and near-infrared range of the electromagnetic spectrum, have been successively investigated at infrared frequencies^5^, and their extension towards the terahertz (THz) region (1 THz=33 cm^−1^=300 μm=4 meV), a spectral range that has seen recently a tumultuous technological and scientific development^6^, is highly desirable. THz research has been oriented either on investigating novel radiation sources based on frequency conversion, optical rectification^7^,^8^,^9^ and relativistic electrons^10^,^11^,^12^,^13^,^14^,^15^, and in studying the properties of plasmon-based systems whose optical properties such as absorption, dispersion and scattering can be engineered at THz frequencies^16^,^17^,^18^. As a matter of fact, the discovery of natural materials showing exotic nonlinear THz properties could set a new agenda in photonic and plasmonic applications of THz radiation.

One of the first observations of a nonlinear THz effect has been achieved in doped semiconductors by means of a THz electric field in the hundred of kV cm^−1^ range^19^,^20^. In GaAs, for instance, free-charge carriers introduced by doping or thermally excited in the conduction band can be accelerated by the THz electric field. When the momentum gain is sufficiently high, carriers are scattered from the bottom of the conduction band into satellite valleys. Here electrons show an increased effective mass with respect to low-lying states, leading to a reduction of carrier mobility and then to an enhanced THz transparency. A similar band-structure effect has been observed in Si and Ge, giving rise to a comparable THz-induced transparency^21^.

Recently, theoretical models have predicted a strong nonlinear THz response of two-dimensional (2D) metallic systems characterized by massless Dirac electrons. Their nonlinear response has been estimated to be higher than massive electron plasma in conventional metals^22^,^23^,^24^. This strong nonlinearity can be qualitatively understood in terms of a simple calculation^23^. Consider charge carriers having a Dirac dispersion 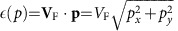, where **p** is the linear momentum and **V**_F_ is the Fermi velocity. Under an oscillating THz electric field *E*(*t*)=*E*_0_ cos*νt*, electrons gain (for zero scattering) a momentum 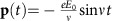. The band velocity (for instance along the *x* direction) can be calculated by the equation 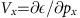 and, for vanishing *p*_*y*_, *V*_*x*_(*t*)=−*V*_F_ sgn(sin*νt*). Therefore, if *n*_D_ is the Dirac surface density and neglecting the statistical distribution of carriers, this corresponds to an intraband Dirac current *J*_D_(*t*)=*en*_D_*V*_F_sgn(sin*νt*)=(4/*π*)e*n*_D_*V*_F_[sin*νt*+1/3 sin3*νt*+1/5 sin5*νt*+....], which contains all odd harmonics *J*_*m*_ (*m*=1, 3, 5, ..), with an amplitude decreasing as 1/*m* (ref. 24). Although both the presence of scattering and the actual statistical distribution of electrons may modify the harmonic intensity ratio as theoretically calculated^25^,^26^,^27^ and experimentally observed in graphene^28^, the previous result still remains valid, and it is exclusively due to the peculiar charge-carrier Dirac energy/momentum dispersion.

Graphene is the first material in which massless Dirac carriers have been predicted theoretically and soon experimentally observed^29^,^30^. Here different experiments have further shown sizable nonlinear optical effects at THz frequency^28^,^31^,^32^,^33^,^34^. However, Dirac electrons have been recently discovered in many other materials such as three-dimensional (3D) Dirac semimetals Cd_3_As_2_ and NaI_3_ (refs 35, 36), giving rise to intensive investigations of their intriguing properties. Perhaps, one of the most important classes of Dirac systems is represented by 3D topological insulators. These materials are quantum systems characterized by an insulating electronic gap in the bulk, whose opening is due to strong spin–orbit interaction, and gapless surface states at their interfaces^37^. Surface states in topological insulators are metallic, characterized by a Dirac dispersion, showing a chiral spin texture^38^,^39^ and protected from backscattering by the time-reversal symmetry. Since their discovery, topological insulators have attracted a growing interest due to their potential application in quantum computing^40^,^41^, THz detectors^42^ and spintronic devices^43^. Linear THz spectroscopy has been applied to 3D topological insulators, in particular on thin films of HgTe and Bi_2_Se_3_, and their both Dirac single particle^44^,^45^,^46^,^47^ and collective (plasmon)^48^,^49^,^50^ excitations versus temperature and applied magnetic field have been observed. However, to our knowledge, THz nonlinear electrodynamics properties have been never investigated on 3D topological insulators so far. In this paper, we fill this gap, reporting on the experimental observation of a strong nonlinear THz absorption in Bi_2_Se_3_ topological insulator thin films. Their electromagnetic response has been studied over seven decades of THz electric field amplitude (from 0.1 V cm^−1^ to 1.5 MV cm^−1^) by combining linear spectroscopy through conventional THz radiation with data achieved at the high-intensity SPARC_LAB linac-based THz source in Italy. This source delivers broadband highly intense THz pulses with femtosecond shaping^12^,^14^. In Bi_2_Se_3_ topological insulator, we observe an induced electromagnetic transparency that increases and eventually saturates at high THz electric fields. This nonlinear THz behaviour is associated with the presence of Dirac electrons onto the surfaces. Indeed, in (Bi_0.9_In_0.1_)_2_Se_3_, a material having the same crystal structure of Bi_2_Se_3_ and showing instead a trivial topology characterized by a gas of massive (Schrödinger) electrons at the surface^51^, we do not observe any nonlinear effect in the same electric field interval.

## Results

### Grown and characterization of Bi_2_Se_3_ thin films

Two thin films of Bi_2_Se_3_ were grown by molecular beam epitaxy on a 0.5-mm-thick sapphire (Al_2_O_3_) substrate. One film had a thickness *t*=120 quintuple layers (QLs), where 1 QL ≃1 nm, the other one *t*=60 QL (refs 52, 53). An additional film doped by In, (Bi_0.9_In_0.1_)_2_Se_3_, with *t*=60 QL, which shows a trivial topology and a massive (Schr*ö*dinger) electron gas with a similar surface density of Bi_2_Se_3_ (ref. 51), was grown on the same substrate for a sake of comparison. Each film was preliminarily characterized by transport and Hall measurements^52^,^53^. Their linear THz response was further investigated by Fourier transform spectroscopy^48^,^49^ (Supplementary Note 1).

### Nonlinear THz measurements

**Figure 1:**
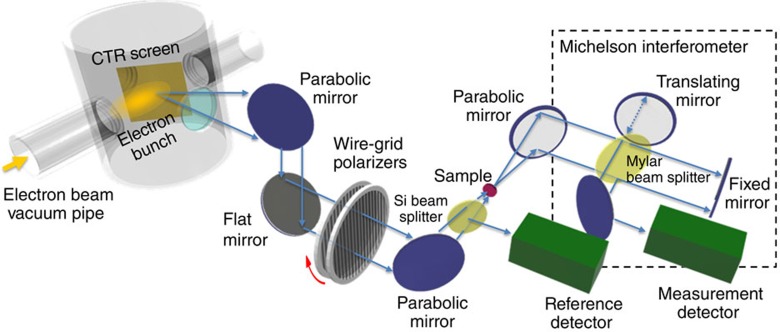
Scheme of experimental set-up at SPARC terahertz source. Ultra-short electron bunches, from the high-brightness photoinjector, interacting with a metallic screen produce highly intense sub-picosecond coherent transition radiation THz pulses. THz radiation (blue arrows), emitted at 90° with respect the electron propagation direction, is transmitted by a *z*-cut quartz window, and collected and collimated by means of an axis-off parabolic mirror. A further flat mirror was used to reflect the THz radiation up to the optical table, where a second off-axis parabolic mirror focalized the THz pulses on film samples. A pair of parallel wiregrid polarizers (QMC Inc.) have been used to tune the amplitude of the THz electric field over four decades: from 1 kV cm^−1^ to 1.5 MV cm^−1^. A further, twin, off-axis parabolic mirror is finally used for illuminating a Michelson interferometer equipped with a GENTEC-EO pyroelectric detector that has been used for measuring the spectrally resolved transmittance. A further GENTEC-EO pyroelectric detector (reference detector) was mounted before the films to implement a differential detection to remove the shot-by-shot fluctuation effects of the SPARC THz source. Integrated transmittances were measured substituting the Michelson interferometer with another GENTEC-EO pyroelectric detector that is mounted just behind the films.

In Fig. 1, the experimental set-up is reported. Highly intense sub-picosecond THz pulses are produced at SPARC_LAB as coherent transition radiation (CTR) emitted by ultra-short (≃120 fs) high-brightness electron bunches^11^,^14^. THz radiation is reflected at 90° with respect to the electron beam direction and transmitted through a *z*-cut quartz window to an off-axis parabolic mirror. This mirror produces collimated radiation that is further reflected by a flat mirror at 45° and finally focalized on the film surface by a third off-axis parabolic mirror. A silicon beam splitter mounted before the films uses a portion of the beam to implement a differential detection system, allowing to remove the shot-by-shot fluctuation effects of the source. Finally, a pair of parallel wiregrid polarizers was used to tune the amplitude of THz electric field over four decades: from 1 kV cm^−1^ to 1.5 MV cm^−1^. We performed both integrated transmittance measurements (through a pyroelectric detector mounted behind the samples that collects the THz intensity transmitted by the film (substrate)) and spectrally resolved ones using a step-scan Michelson interferometer (Fig. 1). Integrated and spectrally resolved transmittance at lower electric fields (from 0.1 to 1 V cm^−1^) was measured by Fourier transform spectroscopy with a conventional mercury lamp. As film transmittances have been normalized to the transmittances of the bare substrate, we have verified that the sapphire substrate response is independent of the amplitude of the THz field. Therefore, the nonlinear THz effects here observed are exclusively due to the Bi_2_Se_3_ topological insulator films.

**Figure 2:**
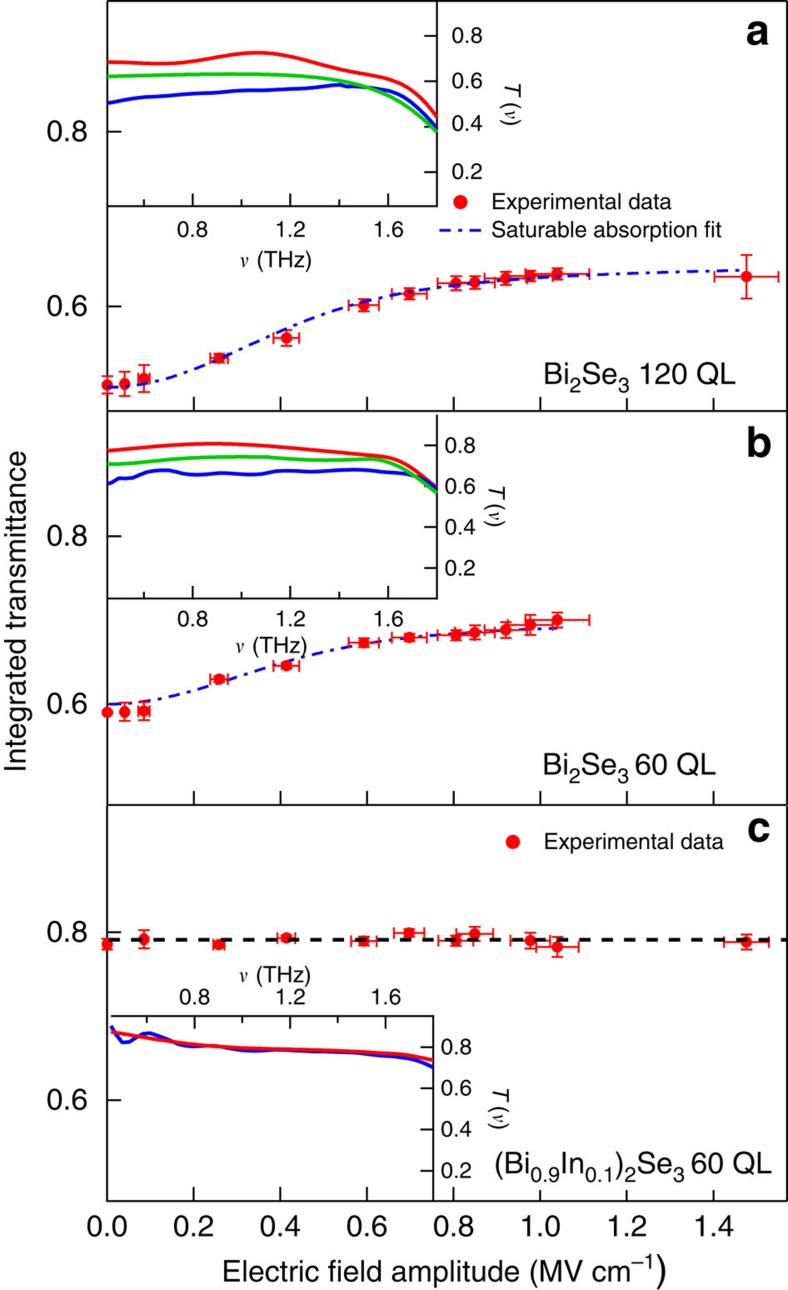
THz nonlinear behaviours of the Bi_2_Se_3_ topological insulator. Integrated transmittance of Bi_2_Se_3_ 120-QL (**a**), Bi_2_Se_3_ 60-QL (**b**) and (Bi_0.9_In_0.1_)_2_Se_3_ 60-QL (**c**) films, respectively, as a function of the incident THz electric field amplitude. Experimental data are represented by red dots. The error bars on the order of 0.5% on both the integrated transmittance and electric field amplitude correspond to the statistical fluctuations of the measured signals averaged over 100 shots of the SPARC THz source. The dashed dotted blue line corresponds to a fit with a saturable absorption model that is described in the main text. Insets: spectrally resolved transmittance curves measured (solid lines) at 1 MV cm^−1^ (red curve), 0.4 MV cm^−1^ (green curve) and 0.1 V cm^−1^ (blue curve) for Bi_2_Se_3_ 120 QL (**a**) and Bi_2_Se_3_ 60 QL (**b**). The spectrally resolved transmittance of (Bi_0.9_In_0.1_)_2_Se_3_ 60 QL at 0.1 V cm^−1^ (blue curve) and 1 MV cm^−1^ (red curve), which are superimposed in the limit of our sensitivity, is shown in the inset of **c**. The slow modulation in the spectrally resolved transmittances are related to a non-perfect compensation of water absorption in the THz range.

Figure 2a,b shows the integrated transmittance at 300 K, normalized to that of substrate versus the THz electric field amplitude *E*_0_ for the 60-QL and 120-QL Bi_2_Se_3_ films. From 0.1 to 50 kV cm^−1^ the integrated transmittance (on the order of 60% for 60 QL and 50% for 120 QL) does not change appreciably. Therefore, this electric field range corresponds to the linear region in which the optical properties of Bi_2_Se_3_ are practically independent of the THz field. For fields above this range, the integrated transmittance follows a monotonous increasing behaviour, indicating an enhancement of transparency. For fields above 1 MV cm^−1^, instead it begins to saturate to a value of 70% (63%) for 60-QL (120-QL) film, corresponding to an enhanced transparency of ∼20% with respect to the value in the linear region.

In the insets of Fig. 2a,b, we show the frequency-resolved transmittance for both the samples at three different electric field amplitudes: 0.1 V cm^−1^ (as measured by a Michelson interferometer coupled with a mercury source), 0.5 and 1 MV cm^−1^ (these last values by the THz source at SPARC_LAB). At the lowest field, both the films show a transmittance that slightly decreases for frequency *ν*→0. This behaviour is the signature of a free electron (Drude) absorption, which is associated mainly with Dirac surface states^46^,^47^, although a contribution from a 2D massive electron gas due to band-bending effects, cannot be ruled out, in particular at room-*T*^54^. Moreover, in the high-frequency part of the transmittance spectrum (∼1.8 THz), one observes a minimum that is related to the presence of the bulk α-phonon absorption superimposed to the Drude term (see Supplementary Note 1, Supplementary Table 1 and Supplementary Fig. 1 for details). Spectrally resolved transmittances are quantitatively similar to the ones measured on films belonging to the same batch in refs 46, 47, 48, 49. By increasing the THz field, transmittances become more flat and, at 1 MV cm^−1^, enhance their value to ∼80%(75%) for the 60 (120)-QL films.

The integrated transmittance as a function of the THz electric field amplitude *E*_0_ can be described by a phenomenological saturable absorption model:


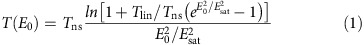


where *T*_lin_ and *T*_ns_ are the linear and the non-saturable integrated transmittances, and *E*_sat_ is the THz electric field saturation value. If one fixes *T*_lin_ and *T*_ns_ from the data (*T*_lin_=0.59% and *T*_ns_=0.69% for 60-QL film, *T*_lin_=0.51% and *T*_ns_=0.65% for 120-QL film), one obtains from the fit (blue point-dashed curves in Fig. 2a,b), comparable values of *E*_sat_ for both the samples: 0.32 MV cm^−1^ for 120 QL and 0.31 MV cm^−1^ for 60 QL, which correspond to a saturation fluence *F* of ∼50 μJ cm^−2^.

The constant value of *E*_sat_ as measured on different thickness (60 and 120 QL) Bi_2_Se_3_ films is a clear signature that the nonlinear absorption in topological insulators is a surface property and does not depend on their bulk characteristics. Moreover, such a saturation fluence is comparable to that measured in doped graphene (*F*=20 μJ cm^−2^)^32^.

To properly assign the nonlinear electromagnetic response observed in Bi_2_Se_3_ to Dirac electrons, we have investigated also the optical response of a (Bi_1−*x*_In_*x*_)_2_Se_3_ film with *x*=0.1. While indium substitution does not change the crystal structure, it induces a quantum phase transition from a topological to a trivial phase for an In content larger than 0.045 (ref. 47). Moreover, the (Bi_0.9_In_0.1_)_2_Se_3_ film shows, due to the band-bending effects, a gas of massive electrons having a surface density (*n*_M_=2.5 × 10^13^ cm^−2^), comparable to the Dirac surface density in Bi_2_Se_3_ (ref. 51). At variance with Bi_2_Se_3_, the integrated transmittance of (Bi_0.9_In_0.1_)_2_Se_3_, which is shown in Fig. 2c, is completely flat over seven decades of the THz electric field amplitude. Moreover, the spectrally resolved transmittances both at 0.1 V cm^−1^ and 1 MV cm^−1^ are superimposed within our experimental sensitivity. These results undoubtedly indicate that the strong nonlinear electromagnetic response previously observed in Bi_2_Se_3_ must be attributed to the 2D gas of Dirac electrons present at the surface of topological insulators.

## Discussion

**Figure 3:**
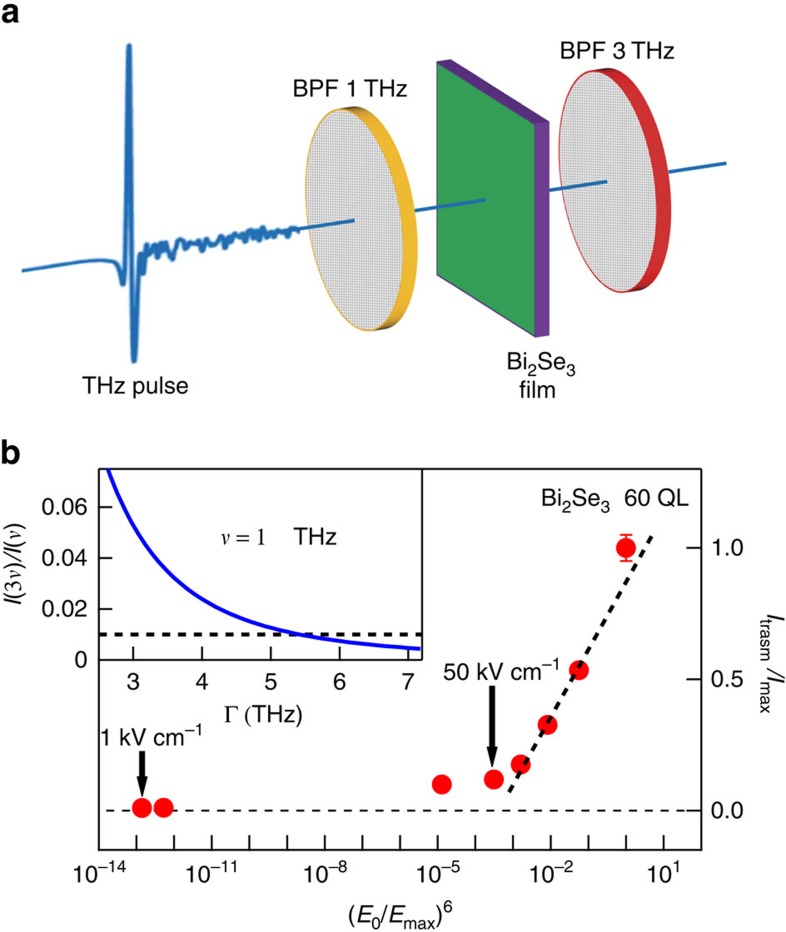
Third harmonic generation in Bi_2_Se_3_ topological insulator. Optical scheme for a third harmonic measurement. A band-pass optical filter (BPF) selects from the broad SPARC THz spectrum a pulse centred at 1 THz and having a full width at half maximum of 0.18 THz. This pulse, with a maximum electric field of *E*_max_=300 kV cm^−1^, illuminates a 60-nm-thick Bi_2_Se_3_ film. The transmitted intensity is collected through a filter centred at 3 THz with a full width at half maximum of 0.36 THz, and finally measured through a pyroelectric detector (**a**). Above nearly 50 kV cm^−1^ the transmitted intensity (normalized to its maximum value) follows a (*E*_0_/*E*_max_)^6^ dependence, suggesting a third harmonic conversion process (**b**). In the inset of **b**, the efficiency 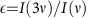 of third harmonic generation (where I(3*ν*) and I(*ν*) are the transmitted intensities of a sample at a frequency 3*ν* and *ν*, respectively) is represented versus the charge-carrier scattering rate Γ. The measured efficiency of 1% is obtained for Γ ∼5.5 THz.

In graphene, the electromagnetic-induced transparency has been interpreted in terms of a combination of the two mechanisms: harmonic generation and a strong decrease of carrier mobility (that is, an increase of carrier scattering rate), due to the opening of new scattering channels for the accelerated carriers^28^,^32^,^33^. To clarify the transparency microscopic mechanism in Bi_2_Se_3_ topological insulator, we have performed a further experiment on a 60-QL-thick film with the aim of looking for a specific harmonic signal. In particular, a pulse centred at 1 THz with a maximum field of ∼300 kV cm^−1^ (selected from the broad SPARC spectrum by a band-pass THz filter, see the Methods section and Supplementary Information) has been used to stimulate the 60-QL Bi_2_Se_3_ film. Let us observe that at 1 THz the SPARC source has its maximum intensity (Supplementary Fig. 2). The transmitted intensity (for the optical scheme see Fig. 3a) was collected through a band-pass filter centred at 3 THz and finally measured through a pyroelectric detector. Both the filters (whose THz response is shown in Supplementary Fig. 3) have a maximum transmittance of ∼80%, and a full width half maximum bandwidth, of 0.18 THz (at 1 THz) and 0.36 THz (at 3 THz). Let us note that the full width half maximum bandwidth of the 3-THz filter is sufficiently broad to capture the third harmonic signal (1→3 THz) in Bi_2_Se_3_ films. By finally varying the incident intensity (that is, the incident electric field) through a couple of THz polarizers, we were able to measure the transmitted intensity from an incident field *E*_0_ of ∼1 kV cm^−1^ to a maximum field of 300 kV cm^−1^. This intensity (normalized to its maximum value in Fig. 3b), first increases smoothly at the low-field values. Around 50 kV cm^−1^ it rapidly grows, scaling with (*E*_0_/*E*_max_)^6^ (black dashed line in Fig. 3b), where *E*_max_=300 kV cm^−1^. Although, from the present measurements, we cannot exclude incoherent contribution to the 3-THz signal coming from supercontinuum generation and hot-carrier emission^27^, its dependence on the sixth power of *E*_0_ is highly suggestive (see for instance ref. 55), of a third harmonic generation (THG) process. In this regard, one can note that 50 kV cm^−1^ corresponds to the field where the electromagnetic momentum gained by electrons is comparable with the Fermi momentum, that is, the threshold at which one expect strong nonlinear effects.

The efficiency of THG process can be measured by the ratio 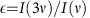, where *I*(3*ν*)/(*I*(*ν*) is the transmitted intensity of a sample at the frequency 3*ν* (*ν*).This ratio at *ν*=1 THz is ∼1% in quite good agreement with graphene^28^. 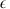 depends on four parameters of the Dirac electron gas: the Fermi velocity *V*_F_, the Fermi energy *E*_F_, the Dirac charge density *n*_D_ and the actual scattering rate of charge-carrier Γ (ref. 26). 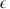 has been calculated theoretically for graphene in ref. 26 and calculation can be extended to Bi_2_Se_3_. Indeed, the first three parameters are well known from transport and photoemission experiments performed on films of the same batch^47^,^52^: *V*_F_=5±1 × 10^7^ cm s^−1^, *E*_F_=380±10 meV and *n*_D_=3±1 × 10^13^ cm^−2^. Therefore, using equation 11 in ref. 26, a strong reduction of the efficiency from the ideal value of 1/3 to ∼6% is already obtained at 300 kV cm^−1^ and 1 THz, if the scattering rate is set to its linear value of 3.1 THz. An efficiency of ∼1% is finally achieved by considering a further field renormalization of the scattering rate. Indeed, 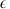 is a rapid decreasing function of Γ (inset in Fig. 3b), and takes a value ∼1% when Γ is ∼5.5 THz. This suggests a quite strong enhancement of the scattering rate of Dirac electrons versus *E*_0_ as already observed for graphene in refs 25, 26, 32.

In graphene, several mechanisms contribute to the scattering of Dirac electrons: electron–electron scattering, short- and long-range impurity scattering, and optical–phonon interaction^32^,^56^. The relative importance of these mechanisms depends on the Fermi energy and temperature. As a matter of fact, to a strong THz pulse corresponds an overall increase of the scattering rate and then a reduction of harmonic conversion efficiency. Although poor information is present about the relative importance of the different scattering mechanisms in Bi_2_Se_3_, one can expect that a similar enhancement of the scattering rate takes place and this renormalization may continue even at higher electric field. Let us observe that taking into account the quite large decreasing of Γ with *T*^48^, one can expect an enhancement of the conversion efficiency at low temperature. Moreover, 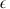 could be further improved by reducing the impurity and defect scattering contributions through a better control of film-growing process.

The electromagnetic response of Bi_2_Se_3_ thin films has been investigated over seven decades of THz electric field: from 0.1 V cm^−1^ by means of conventional Fourier transform THz spectroscopy to 1.5 MV cm^−1^ using the linac-based SPARC_LAB THz source. We observed a strong reduction of the absorption of Bi_2_Se_3_ topological insulator for an increasing THz field that determines an electromagnetic-induced transparency in this material.

The induced transparency is determined only by the surface states of Bi_2_Se_3_ as films with different thickness shows exactly the same THz behaviour. Moreover, a similar experiment performed on a (Bi_0.9_In_0.1_)_2_Se_3_ film, which presents a trivial topological phase characterized by a gas of massive electrons, shows an absorption that does not depend on the THz field amplitude. This demonstrates that the strong nonlinear effects in Bi_2_Se_3_ are driven by massless Dirac electrons at the surface.

Incoherent emission processes at high frequency may contribute to the induced transparency. However, as a consequence of a nonlinear stimulation at 1 THz, the transmitted signal at 3 THz clearly scales with the sixth power of the electric field amplitude. This result suggests a THG process. The efficiency of harmonic generation is on the order of 1% at variance with the nominal value of 1/3. This strong renormalization is mainly determined by the charge-carrier scattering rate. Therefore, the electromagnetic transparency in Bi_2_Se_3_ is generated by two concomitant effects: an intrinsic harmonic generation process and an extrinsic mobility reduction. Then, one could increase the harmonic conversion efficiency by working at low temperature and further improving the film quality.

In conclusion, the possibility to control light by light in the THz regime is an actual subject of intense study to implement compelling applications in THz technology, such as ultrafast THz tabletop sources, quantum cascade lasers and ultrafast THz communications based on optical bistability. In this regard, the strong nonlinear THz properties observed in Bi_2_Se_3_ Dirac material could open promising perspectives in the tumultuous field of THz technologies.

## Methods

### Topological insulator films

The high-quality (Bi_1−*x*_In_*x*_)_2_Se_3_ thin films were prepared by molecular beam epitaxy using the standard two-step growth method developed at the Department of Physics and Astronomy Rutgers, The State University of New Jersey^52^,^53^. The 10 × 10-mm^2^ Al_2_O_3_ substrates were first cleaned by heating to 750 °C in an oxygen environment to remove organic surface contamination. An initial 3 QLs of Bi_2_Se_3_ were deposited on the substrates at 110 °C, which was then followed by heating to 220 °C helping further to achieve the target thickness. The crystallization of the films during the growth was monitored by reflection high-energy electron diffraction. The Se:(Bi/In) optimal flux ratio was 10:1 for the deposition. A pre-control Bi/In flux ratio was performed to achieve the desired In concentration. Once the films were cooled, they were removed from the vacuum chamber, and vacuum-sealed in plastic bags within 2 min, then shipped to the University of Rome.

### High-field THz generation and measurements

The ultra-relativistic electron bunches 650 pC charged with a time duration of ≃120 fs and 10 Hz of repetition rate were used to generate nearly single-cycle THz pulses as coherent transition radiation at SPARC_LAB^12^,^14^. The THz electric field in the focal point was estimated in two ways. The first estimate consists in using the nominal sensitivity of the pyroelectric detector (140 kV W^−1^) that has been experimentally tested at 970 GHz through a Schottky Diode (Virginia Diode) emitting a power of 1 mW. In the second estimate, we calculated the THz electric field in the focal point produced by the CTR SPARC source by a THz transport simulation program^57^. In this calculation, we take into account the actual (finite) size of CTR target, the actual transmittance of the *z*-cut quartz window and the optical properties of the propagation optics. Both the methods provide comparable THz electric fields in the focal point having a highest THz field amplitude of 1.5 MV cm^−1^.

The THz radiation coming from the CTR source was separated in two beams by a high-resistivity Si beam splitter at 45°(Fig. 1). The transmitted beam propagates towards the films, while that reflected one towards a pyroelectric detector. The THz-integrated spectra were measured placing just behind the films a pyroelectric detector, meanwhile the THz spectra have been measured by a step-scan Michelson interferometer, having a 24-μm Mylar pellicle beam splitter. All radiation channels were equipped with THz-I-BNC GENTEC-EO pyroelectric detectors.

We used a differential detection technique to reduce the shot-to-shot fluctuations of the SPARC THz source. In particular, both the integrated signal and the spectrally resolved one were normalized to the signal as measured by the reference pyroelectric detector. This technique provides an error bars on the transmittance (both integrated and spectrally resolved) on the order of 0.5%.

The THz filters used in the harmonic detection experiment have been acquired from Tydex (http://www.tydexoptics.com). Their optical response controlled by a Michelson interferometer are reported in Supplementary Fig. 3. The THz radiation transmitted by the series 1-THz filter, 60-QL film and 3-THz filter has been finally collected using a THz-I-BNC pyroelectric by GENTEC-EO.

## Additional information

**How to cite this article:** Giorgianni, F. *et al*. Strong nonlinear terahertz response induced by Dirac surface states in Bi_2_Se_3_ topological insulator. *Nat. Commun.* 7:11421 doi: 10.1038/ncomms11421 (2016).

## Supplementary Material

Supplementary Information Supplementary Figure 1-3, Supplementary Table 1, Supplementary Notes 1-2 and Supplementary References
