# Supplementary material for: Strong nonlinear terahertz response induced by Dirac surface states in Bi2Se3 topological insulator
Source: Nat Commun. 2016 Apr 26;7:11421. doi: 10.1038/ncomms11421 (PMC4853424; doi:10.1038/ncomms11421)
Supplement: Supplementary Information — Supplementary Figure 1-3, Supplementary Table 1, Supplementary Notes 1-2 and Supplementary References [file ncomms11421-s1.pdf]

Supplementary Figure 1. Transmittances of $(Bi_{x-1}In_x)_2Se_3$ films in the terahertz range at room temperature. a): transmittance of the x=0, 120 QL film; b) transmittance of the x=0, 60 QL film; c) transmittance of the x=0.1, 60 QL film; Drude-Lorentz fits (Supplementary Equation 2) are indicated with dashed blue points.

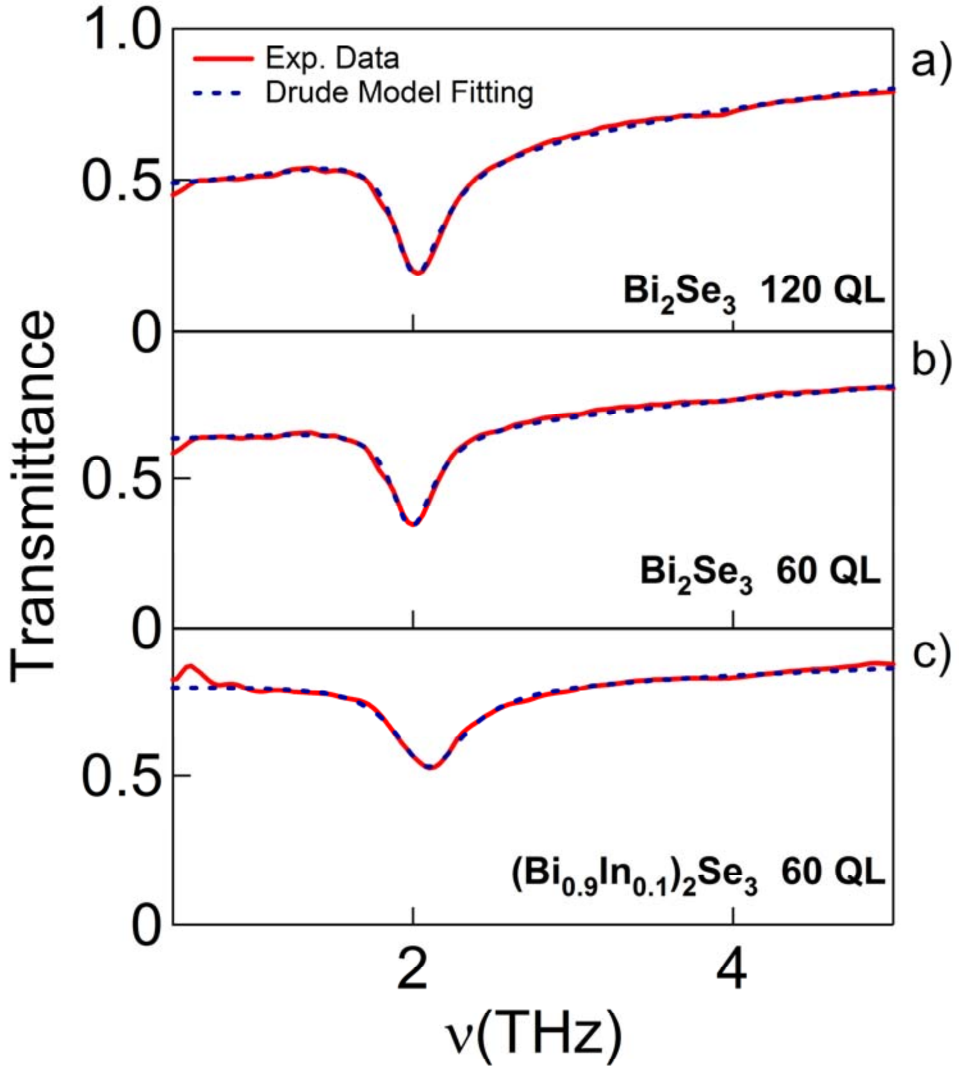

Supplementary Figure 2. a) Transverse profile of the Coherent Transition Radiation on the samples focal plane at 1 THz: experimental data (red line) and numerical simulation by THz Transport (dotted-blue line); CTR transverse spatial distribution (inset); b) electric field spectral distribution of CTR in the THz range evaluated in the focal plane.

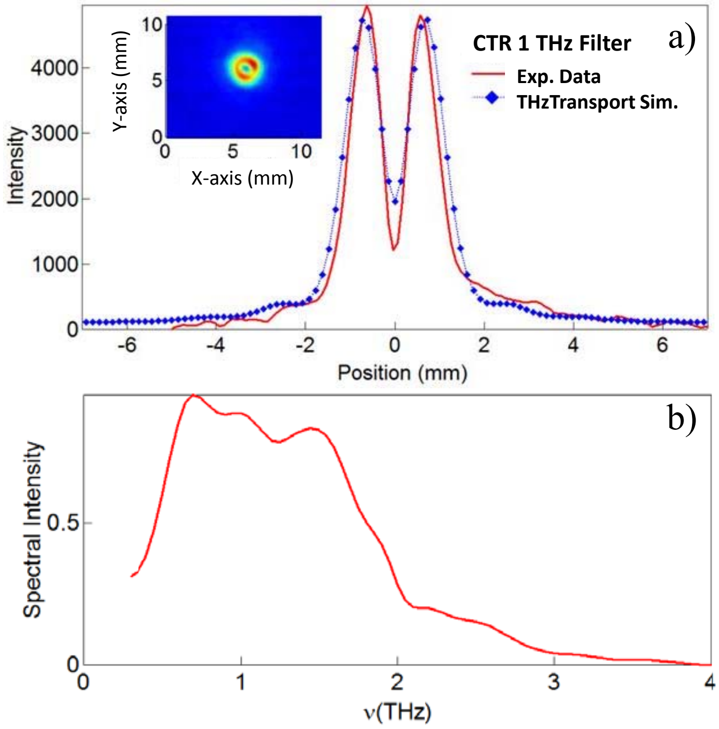

Supplementary Figure 3. Transmittance of band-pass THz filters centered at 1 THz and 3 THz with a bandwidth (Δν) of 0.18 THz and 0.36 THz, respectively.

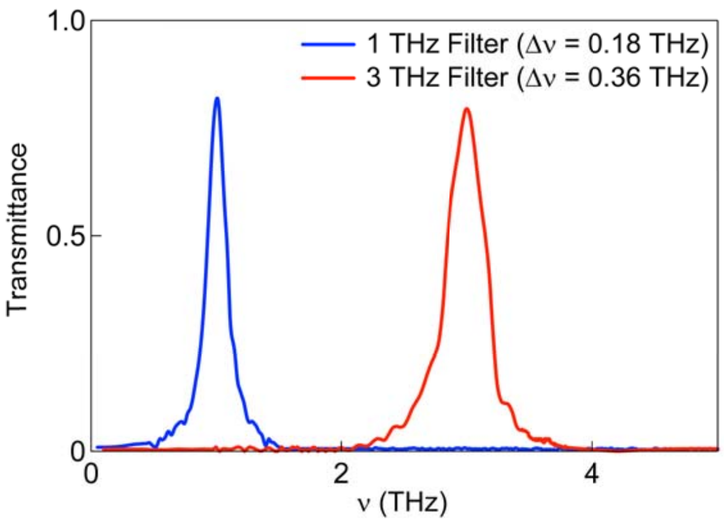

Supplementary Table 1. Drude-Lorentz parameters as extracted from the THz transmittance at room temperature for both Bi_2Se_3 and $((Bi_{0.9}In_{0.1})_2Se_3)$ films.

Films	ν_p (THz)	Γ (THz)	S_α (THz)	ν_p (THz)	Γ_α (THz)	ϵ_∞
Bi_2Se_3 120 QL	41.7	2.6	19.5	1.98	0.28	30
Bi_2Se_3 60 QL	57.2	3.1	19.3	1.98	0.23	30
$(Bi_{0.9}In_{0.1})_2Se_3$ 60 QL	49.8	5.8	19.7	2.11	0.44	30

Supplementary Note 1. THz measurements in linear regime and Drude-Lorentz Fit

Fourier Transform Infrared (FT-IR) Spectroscopy transmission measurements have been performed with a Bruker Michelson interferometer coupled to a 4.2 K silicon bolometer in order to monitor the linear response of Bi_2Se_3 films. The transmittance T(ν) of Bi_2Se_3 thin films has been measured with respect to that of a bare sapphire substrate and can be described in terms of the Tinkham thin-film formula [1]

$$T(\nu) = \frac{1}{|1 + \tilde{G}(\nu)\frac{Z_0}{n_{sub}+1}|^2} \,, \tag{1}$$

where

$$\tilde{G}(\nu) = \Big[\frac{-\nu_p^2}{i\nu - \Gamma} - \frac{i\nu S_\alpha^2}{-\nu(\nu + i\Gamma_\alpha) + \nu_\alpha^2} + i\nu(\epsilon_\infty - 1)\Big]\epsilon_0 t \tag{2}$$

is the complex conductance, Z_0 is the impedance of free space, n_{sub}=10.5 is the refraction index of sapphire in the THz range, which does not depend on frequency.

In the THz range $\tilde{G}(\nu)$ can be described in terms of a Drude-Lorentz (DL) model through the sum of three contributions [2]: a Drude term describing the Dirac (massiveless) quasi-particle response and two Lorentz oscillators related to the bulk α- and β-phonon absorption. Due to its small-intensity the β phonon can be observed only at low-temperature and it has been not included in the fit at 300 K. The plasma frequency ν_p and the scattering rate Γ of the Drude term as well as the amplitude S_α, central frequency (ν_α) and width (Γ_α) of the α-phonon are reported in Supplementary Table 1 for the three films here measured. The experimental transmittances and fit curves are shown instead in Supplementary Fig. 1.

Supplementary Note 2. Details of the SPARC LAB Terahertz Source

The THz nonlinear absorption experiment has been performed at the SPARC_LAB test facility [3], INFN National Laboratory of Frascati (INFN-LNF, Italy). The facility consists of a photo-injector, which delivers ultra-short high brightness electron beams to drive the THz emission [4, 5].
Ultra-short high intensity THz radiation pulses are produced through the coherent transition radiation (CTR) mechanism. Transition radiation (TR) occurs when a relativistic particle crosses the boundary between media with different

electrodynamics properties. The TR becomes coherent when the emitted wavelength exceeds the longitudinal size of an electron bunch. In this regime all electrons in the bunch emit in phase and the total intensity grows with N^2, where N is the number of electrons in the bunch. To generate CTR in the THz range, the first accelerating section of the SPARC_LAB linear accelerator is used to longitudinally compress the bunch to sub-ps duration through the velocity bunching technique [6]. The compression scheme used in this experiment provides electron beam energy of about 120 MeV with bunch duration of 120 fs and a bunch charge of nearly 650 pC [4]. In these experimental conditions we have a maximum energy per pulse of about 40 μJ and an associated electric field of nearly 1.5 $MVcm^{-1}$.
The experimental setup consists of an aluminum-coated silicon screen to produce CTR; the screen is placed at 45° with respect to the electron beam direction to reflect the CTR out of the vacuum chamber (see Fig.1 in the main text) [5]. THz radiation is then transmitted through a z-cut quartz window to an off-axis parabolic mirror. This mirror produces collimated radiation which is further reflected by a flat mirror at 45° and finally focalized onto the film surface by a third off-axis parabolic mirror with a focal length of 100 mm. Supplementary Figure 2a shows the transverse intensity profile as measured through a THz camera (Spiricon Camera III, Ophir) in the focal plane of the parabolic mirror. Transverse profile measurements have been compared to THz Transport code calculations, which permit to evaluate the transport characteristics along the optical path. By taking into account all optical elements along this path, the numerical calculation (blue solid points) is in very good agreement with experimental data (red curve) (Supplementary Figure 2a).
The spectral properties of the THz radiation have been measured through a step-scan Michelson interferometer equipped with a 12 μm thick Mylar beamsplitter and pyroelectric (GENTEC-EO) detectors. The intensity in the focal point has been varied through a pair of parallel wiregrid polarizers (QMC Inc.), mounted before the last focalizing parabolic mirror. An example of the measured THz spectrum is shown in Supplementary Fig. 2b. The electric field in the focal point has been estimated in two different ways. In the first case through the detector sensitivity (140 kV W^{-1}), we have calculated the total power, which is then transformed in electric field values by knowing the focal point size. The nominal detectivity has been tested with the same THz optical set-up at 970 GHz (where the SPARC_LAB THz source shows the maximum intensity) by measuring the well-known intensity of a (triplicate) Gunn-diode from Virginia-Diode. This kind of calibration has been performed for all detectors here used. In the second case we have calculated the emitted intensity at the focal point through THz Transport code by taking into account the optical response of all optical elements before the samples. Both methods provide well consistent electric field values, which have been used in Fig. 2 of the main text.
We performed both integrated transmittance measurements (just placing a pyroelectric detector behind the films) and spectrally resolved ones through the Michelson interferometer (Fig.1 in the main text). A further pyroelectric detector was mounted before the films to implement a differential detection to remove shot by shot fluctuation effects of the SPARC_LAB THz source.
The experiment, ables to detect a harmonic generation signal (see main text), has been performed through band-pass filters from Tydex Company. They have a maximum transmittance (shown in Supplementary Figure 3) on the order of 80%. The bandwidth (FWHM) is $\Delta\nu$= 0.18 THz (0.36 THz) for the 1 THz (3 THz) filter. The THz radiation transmitted by the series 1 THz filter, 60 QL Bi_2Se_3 film and 3 THz filter has been finally collected by a GENTEC-EO pyroelectric detector.

Supplementary References

[1] Glover III, R. E. and Tinkham, M., Conductivity of Superconducting Films for Photon Energies between 0.3 and 40 kTc , *Phys. Rev. Lett.* **108**, 243 (1957).
[2] Aguilar, R. V. et al., Terahertz response and colossal Kerr rotation from the surface states of the topological insulator Bi_2Se_3, *Phys. Rev. Lett.* **108**, 087403 (2012).
[3] Ferrario, M. et al., SPARC_LAB present and future, *Nucl. Instrum. Methods Phys. Res. B* **309**, 183-188 (2013).
[4] Chiadroni, E. et al., The SPARC linear accelerator based terahertz source, *Appl. Phys. Lett.*, **102** (9), 094101 (2013).
[5] Chiadroni, E. et al., Characterization of the THz radiation source at the Frascati linear accelerator, *Rev. Sci. Instr.* **84**, 022703 (2013).
[6] Ferrario, M. et al., Experimental Demonstration of Emittance Compensation with Velocity Bunching, *Phys. Rev. Lett.* **104**, 054801 (2010).
